# Embedding health policy and systems research into decision-making processes in low- and middle-income countries

**DOI:** 10.1186/1478-4505-11-30

**Published:** 2013-08-08

**Authors:** Adam D Koon, Krishna D Rao, Nhan T Tran, Abdul Ghaffar

**Affiliations:** 1 Department of Global Health and Development, London School of Hygiene and Tropical Medicine, 15-17 Tavistock Place, London, WC1H 9SH, UK; 2 Public Health Foundation of India, 4 Institutional Area, Vasant Kunj, New Delhi, 110070, India; 3 Alliance for Health Policy and Systems Research, World Health Organization, 20 avenue Appia, Geneva, 1211, Switzerland

**Keywords:** Embeddedness, Evidence-informed policy-making, Health policy and systems research, Low- and middle-income countries

## Abstract

Attention is increasingly directed to bridging the gap between the production of knowledge and its use for health decision-making in low- and middle-income countries (LMICs). An important and underdeveloped area of health policy and systems research (HPSR) is the organization of this process. Drawing from an interdisciplinary conception of embeddedness, a literature review was conducted to identify examples of embedded HPSR used to inform decision-making in LMICs. The results of the literature review were organized according to the World Health Organization’s Building Blocks Framework. Next, a conceptual model was created to illustrate the arrangement of organizations that produce embedded HPSR and the characteristics that facilitate its uptake into the arena of decision-making. We found that multiple forces converge to create context-specific pathways through which evidence enters into decision-making. Depending on the decision under consideration, the literature indicates that decision-makers may call upon an intricate combination of actors for sourcing HPSR. While proximity to decision-making does have advantages, it is not the position of the organization within the network, but rather the qualities the organization possesses, that enable it to be embedded. Our findings suggest that four qualities influence embeddedness: reputation, capacity, quality of connections to decision-makers, and quantity of connections to decision-makers and others. In addition to this, the policy environment (e.g. the presence of legislation governing the use of HPSR, presence of strong civil society, etc.) strongly influences uptake. Through this conceptual model, we can understand which conditions are likely to enhance uptake of HPSR in LMIC health systems. This raises several important considerations for decision-makers and researchers about the arrangement and interaction of evidence-generating organizations in health systems.

## Background

As health systems have become more complex and public demands for accountability have increased, the salience of health system performance has grown [1]. The current international emphasis on evaluating performance has positioned health policy and systems research (HPSR) as an important vehicle for promoting evidence-informed decision-making [2]. Recent work has elucidated how unique organizational arrangements can facilitate this exchange [3,4].

In this study, we explore the “embeddedness” of HPSR in decision-making processes in health. We develop a conceptual model that identifies the organizational characteristics that facilitate the embedding of HPSR into decision-making and their interaction with decision-makers in low- and middle-income country (LMIC) health systems. In identifying dimensions of embeddedness, this work raises important considerations for decision-makers, planners, and researchers alike.

The term “embeddedness” has a long history in the social sciences. The concept can be traced to the work of Karl Polanyi, who, in 1957, wrote that “the human economy… is embedded and enmeshed in institutions, economic and non-economic. The inclusion of the non-economic is vital” [5]. This idea of embeddedness, or “social embeddedness”, as it is often referred to, represents an organization’s and/or individual’s connection, relationship, and/or position, within a social network [6]. The term is also associated with the idea of social capital that gained credence in the early 1990’s [7]. Nevertheless, embeddedness assumes many forms, as manifest by its assorted use in sociology, anthropology, political science, public administration, and economics. It has been used to describe electronic social networks [8], engagement of immigrants in politics [9], consumption trends in the agricultural sector [10], as well as the performance of various health agencies in the public sector [11,12].

Due to the complex stakeholder environment, social network analysis provides a useful way of conceptualizing embeddedness in LIMC health systems. According to Huang and Provan [10], the degree of embeddedness of an organization refers to its structural position in an organizational network. The greater its embeddedness or centrality in an organizational network, the greater is its connectivity with other networked organizations and the more immersed the organization is in the flow of information and resources than non-central organizations [12,13]. In their study of a health and human services network, Provan et al. found that embedded organizations possessed several desirable qualities [11]. For one, they were more influential. Influence, in this case, incorporated an embedded organization’s stance, recommendations, or actions being considered when other organizations within the network made important decisions. Embedded organizations were also found to be trustworthy and have strong reputations. Organizations that reliably delivered on their commitments to other actors in the web of exchanges were considered trustworthy. Similarly, organizations perceived to be performing at a high level and producing quality outputs for others within its domain were said to have a strong reputation. These qualities may in part account for an embedded organization’s ability to wield power within and outside the network. Another important characteristic was that embedded organizations increased the performance of the network as a whole. Further, these qualities of organizational embeddedness tended to strengthen as the network matured [13].

Although similar network analyses have been used in various research studies [14-17], to the best of our knowledge this methodology has not been used in assessing the embeddedness of HPSR organizations or any type of organizational arrangement in LMICs.

## Methods

First, literature from various disciplines was consulted to develop the concept of organizational embeddedness. Next, we hypothesized that the quantity, quality, and relevance of HPSR, as well as legislation governing its use would influence the extent to which HPSR was embedded into decision-making in LMICs. We evaluated this hypothesis by conducting a thorough literature review and organizing our findings by health system functions. Finally, we designed a conceptual model to reflect our new understanding of embedded HPSR organizations in LMICs.

Inclusion and exclusion criteria were formulated to account for the abstract nature of research on the decision-making process in health policy, processes of knowledge translation, and generation of research for practical applications in health. We also assessed the growing body of literature around barriers and facilitators to research utilization in health policy [2,18-22]. We included original HPSR articles and review articles that matched our search criteria, were explicitly conducted in or focused on LMICs, and incorporated some aspect of the processes above. Commentaries, editorials, dispatches from the field, news articles, studies conducted in high-income countries, and non-health articles were excluded from the review.

We used a generous time frame, grey literature, and combinations of eleven search terms to account for a perceived paucity of HPSR from LMICs. The following electronic databases were searched up to December 2011 (inclusive): PubMed-Medline (1965–2011); EBSCO Global Health (1973–2011), and Global Health Archive (1910–1983). Additionally, Google and Google Scholar search engines were used to identify sources, such as reports, book chapters, and government documents not included in the electronic databases. Search terms were developed *a priori* by ADK and KDR and included “policy makers”, “decision makers”, “evidence-based policy”, “evidence-based policy-making”, “policy process”, “research to policy”, “embeddedness”, “embedded research”, “social embeddedness”, MeSH “developing countries”, MeSH “low income populations”, and “low- and middle-income countries”. Lastly, we included references cited in relevant studies.

Since health systems are characterized by a diversity of institutions and activities, we organized our data according to the World Health Organization’s (WHO) Health Systems Framework [23]. This well-established framework was conveniently selected from a number of different frameworks [24] for its simplicity and acceptability by the wider global health community as opposed to its analytical value. Likewise, we used the definition of health systems defined by WHO in 2000 as “all the activities whose primary purpose is to promote, restore, or maintain health” [1]. This broad definition is compatible with the concept of embeddedness in that it allows for a multiplicity of actors, interests, and relationships to be characterized within LMIC health systems. With the WHO framework and definition of health systems as our guide, we identified various knowledge-translation pathways and organizational embeddedness with respect to service delivery, health workforce, information, medical products (drugs, vaccines, and devices), financing, and leadership and/or governance. While there is sufficient evidence to warrant this type of classification, there is some degree of overlap with several studies. For example, adoption of a certain course of treatment for malaria could be included in the medical products, service delivery, or even the governance realm. Therefore, this categorization is by no means absolute and judgment was made through consultation between the reviewing author (ADK) and at least one additional author when ambiguity arose. Also, note that we are assuming that decision-makers use research for making decisions. While this may be true of some countries or some health system building blocks within countries, it is unlikely to be true of all contexts.

Descriptive information was extracted from selected articles by ADK. All authors agreed on extraction of the following information from each article: year, location, representative health system building block (see below), knowledge transfer process described, and characteristics of evidence-generating organizations. Articles were excluded if this information was not explicitly reported. Authors consulted with each other when ambiguities arose between papers. KDR repeated the search for verification. Also, snowballing was employed whereby relevant citations from included studies were pursued and included if appropriate. All authors discussed the descriptive information generated from the review, compared it to the original hypothesis, and collaboratively packaged the findings into a conceptual framework that reflected the configuration and attributes of embedded HPSR organizations in LMICs.

## Results

There is a small, but growing volume of literature on HPSR entering into the decision-making sphere. A total of 83 articles were returned from our initial search. Applying our exclusion criteria reduced the number of articles to 55. These abstracts were reviewed in full and another 37 items, including several reports and policy documents, were identified through related citations and via the grey literature. Thus, the total number of articles included in this review was 92. Articles ranged from 1992–2011 (1992–1999: n = 6; 2000–2009: n = 63; 2010–2011: n = 23). Many articles broadly focused on strengthening knowledge translation in several LMICs. Still, nearly half of the articles (n = 44) pertained more directly to one of the health systems building blocks. Some of the strongest examples are described below.

### Service delivery

Several studies (n = 14) have examined the diverse group of actors involved in decision-making around service delivery. These studies indicate important differences in who informs the process by which health services are delivered. Lobby groups, champions, and the leadership of national, regional, and international research and policy networks were paramount in inserting research into the policy process for health care delivery in Mozambique, South Africa, and Zimbabwe [25]. Research to inform planning of various service delivery mechanisms also came from outside the Ministry of Health (MoH) in Kenya and Mexico [26,27]. Within the service delivery block, a great deal of research-informed policy focuses on vertical programs; this tends to draw from a number of different sources. In Uganda, for example, international advisory groups, academics, non-governmental organizations (NGOs), and other peripheral organizations generated disease-specific research, which policy-makers used to base their decisions about several infectious diseases [28]. Unlike Uganda, Peru used a very small set of external actors to evaluate research generated from federal research bodies in reforming malaria treatment policy [29]. Policy may also be formulated despite sound evidence against it. Consider Thailand where, in the face of a quasi-federal organization’s research against scaling-up antiretroviral therapy, a powerful policy network of non-state (NGO and civil society) actors successfully lobbied for the program [30]. Several other important factors were responsible for launching this policy; however, this example illustrates some of the complexities encountered during the process of crafting health policy in LMICs. Lastly, it should be noted that considerable variation was found within the services delivery block and the extent to which decision-making for vertical programs in a given country is indicative of decision-making for other health services is not clear from these studies.

### Medical products (vaccines, drugs, medical devices, etc.)

Of the six building blocks, literature from the medical products block (n = 9) demonstrates some of the clearest pathways through which HPSR can flow directly into policy. It is also populated by an interesting set of institutionalized, sophisticated HPSR organizations. Two studies from Asia describe the sources as well as the users of evidence in crafting policy around drugs, medical devices, and diagnostics [31,32]. In India, Pakistan, Malaysia, Philippines, Thailand, South Korea, and Taiwan, researchers have described large federal bodies responsible for the production of evidence to support policy decisions; this may or may not fall under the purview of the MoH. According to the authors, one institution may govern the entire research production and utilization process in some countries. In others, this is not the case. For example, Taiwan produces evidence for market approval of drugs and medical devices from seven different government bodies, only three of which actually use the information. In all seven countries, legal frameworks are in place to regulate the flow of information from research to policy for medical technologies.

Vaccine policy is interesting for several reasons. First, several different types of evidence are frequently used to inform the debate. Second, many countries have Immunization Technical Advisory Groups for vaccine policy; these vary in composition but usually consist of MoH staff, scientists, and other experts [33]. Third, donors and technical agencies (such as WHO, GAVI, and UNICEF) have a strong influence over decision-making in LMICs. In fact, in some countries, decision-makers have indicated that some of the principal sources of evidence to craft vaccine policy are often WHO guidelines or position papers [34].

The literature on Essential Medicines or National Drug Policies suggests that the pathway from research to policy is similar to that of vaccines. Like vaccine policy, in Mali and Laos, national commissions, composed of an intersectoral set of experts, inform drug policy. In Mali, researchers used evidence from the peer-reviewed literature, technical reports from international organizations, and other country experiences [35]. In Laos, historically, little research has been used by decision-makers despite the efforts of highly capable health research bodies within the country [36]. In fact, in both Mali and Laos, decision-makers indicated that other concerns were given equal, and sometimes more, weight than scientific evidence.

### Health information systems

Few studies (n = 6) provide evidence on the pathways by which other country experiences, technical assistance, or research influences the policy process for health information systems. In Sri Lanka, Hornby and Perera described the challenge of developing process indicators and installing performance management strategies without health information systems or research from other countries to aid their efforts [37]. In Tanzania, the government benefitted from costing analyses generated by external international researchers in order to inform their experimentation with health information systems technology [38]. Gething et al. report Kenyan efforts to develop health information systems [39]. The authors also present statistical techniques to compensate for imperfect national data, which is a major barrier to evidence-based decision-making in Kenya. Another useful example of a peripheral actor supporting the development of information infrastructure is WHO’s Integrated Disease Surveillance and Response program [40]. Some countries have even used certain aspects of this to form their own Integrated Disease Surveillance Units [41]. For many LMICs, there exists a need to develop basic data collection facilities and an information workforce.

### Health workforce

No studies directly matched our search criteria for the health workforce block; however, we found a loosely related study on devolution in Mali [42], reports from regional WHO bodies [43], and a systematic review on policy options for human resources for health (HRH) [44]. The small amount of evidence may be due to the fact that the health workforce traditionally has been seen as an administrative issue of recruitment, cadre establishment and training, transfers, and postings. The Joint Learning Initiative’s 2004 report on “Human Resources for Health: Overcoming the Crisis” and WHO’s 2006 World Health Report “Working Together for Health,” only recently drew attention to the global HRH crisis [45,46]. There is little research that demonstrates the way HPSR has been used to influence health worker retention in rural areas, curb the flow of qualified health personnel across borders and sectors, harness the potential of task shifting, and improve health worker performance [44]. Also decision-makers often lack basic statistics about the size, composition, and distribution of health workers within their own countries [47]. WHO has attempted to improve this situation through publication of guidelines [48] and WHO regional bodies have overseen development of health workforce observatories [43]. While HRH has emerged as a growing field of research, little evidence suggests that HPSR is currently being used to inform decision-making in LMICs.

### Financing

In five studies, HPSR was used by a technical advisory group, research institution, or high-level task force to influence decision-making for health financing. For example, in the mid-1990’s, South Africa and Zambia embarked on ambitious financing reform in the health sector [49]. The extent to which reform efforts were informed by HPSR resulted largely from interactions of working groups with, in some cases, more powerful actors in the political realm [50,51]. Similarly, the formation of Ghana’s national health insurance scheme created clashes between political elites and technical experts [52]. In contrast, a research institute in Thailand was the guiding force behind an ambitious national health insurance scheme during national elections in 2001 [51]. The (Thai) Health Systems Research Institute was created in 1991 as a publicly-funded, autonomous research organization with the mandate of providing policy-relevant health systems research. Though it operates largely outside of the MoH, the health minister chairs the institute’s governing board. Thailand’s successful insurance scheme can be attributable to investment in human resources for health research, which started 10 years prior to the actual reform measure, was maintained by regular input with key policy-makers in the MoH, and involved several other external actors to force the issue onto the policy agenda during a key period of political transition [53]. Hence, the complicated nature of financing in healthcare necessitates the technical input of various experts, such as an intersectoral working group, technical advisory committee, or research institutions. The very structure of this technical assistance and how it interacts with larger socio-political forces often plays a substantial role in the execution of successful policy initiatives.

### Governance/leadership

Nine articles matched our search criteria for the governance and leadership block. Mexico and Thailand provide the clearest examples of linkages between embedded evidence-generating organizations and decision-makers to inform health sector governance. In the early 2000’s, Mexican decision-makers drew from multiple sources, namely international academic institutions, free-standing publicly-funded institutions, and evidence generated from within the MoH to guide the process of comprehensive healthcare reform [27,54]. Similar to Thailand, Mexico installed a national health insurance scheme to curb regressive out-of-pocket expenditures in healthcare. Also, both Mexico and Thailand relied heavily on HPSR organizations that were created with a public mandate 10–20 years prior to embarking on reform [53,55]. Furthermore, both organizations enjoy direct contact with the Ministers of Health on a regular basis [56]. Thus, two of the most widely cited examples of effective healthcare reform initiatives have utilized HPSR generated from reputable organizations with strong political connections and the capacity to generate useful evidence. Also, both countries relied, to different extents, on legislative frameworks to direct the process [53,54].

The literature suggests that only in unique circumstances has evidence been used to inform governance in other LMICs. Between 2001 and 2006, a government program in the Indian state of Karnataka was established for the sole purpose of fighting generalized poor governance and systemic corruption [57]. In Vietnam and the Solomon Islands, peripheral actors helped to facilitate the creation of national mental health policy [58,59]. In another conflict-affected fragile state, East Timor, the fledgling government began an arduous process of reconstructing the national health system by commissioning research and transferring stewardship responsibility from humanitarian aid organizations to the expanding national government [60]. This underscores the unique circumstances afflicting some countries prior to the development of formal institutions.

## Discussion

The findings of the literature review caused us to reject our original hypothesis and re-calibrate our understanding of embedded HPSR. Our original hypothesis was that the quantity, quality, and relevance of HPSR, as well as legislation governing its use would influence the extent to which HPSR was embedded into decision-making in LMICs. This hypothesis is focused primarily on the characteristics of HPSR, but what the literature suggested was that for HPSR to be embedded, it must be generated from an embedded organization. Therefore, the first shift was from the content of HPSR to the organizations that produce it. Next, it was clear that the quantity and quality of HPSR generated by a given organization mattered much less than the extent to which that organization was connected to others, as well as to decision-makers. Likewise, if an organization was determined to be reputable, then it was likely to have been producing relevant HPSR of reasonable quality and in sufficient quantity. Finally, legislation proved to be an important part of the process, but not necessarily as a dimension of embedded HPSR organizations, but rather as one of several key intermediaries of the decision-making environment. Thus, we developed the conceptual framework below with our new interpretation of embeddedness.

### Conceptual framework for embeddedness in health research

Several historical, social, and political forces converge to create context-specific pathways through which HPSR enters into the decision-making environment. These pathways are regulated by organizational arrangements that influence the interaction between decision-makers and producers of HPSR, including HPSR divisions or expert committees within the MoH, publicly-funded external organizations, and an increasingly complex array of privately-financed external institutions. Depending upon the policy under consideration, decision-makers may call upon an intricate combination of actors within this configuration. For example, in some countries decision-makers convene a task force composed of researchers prior to undertaking a major policy endeavour, like formulating a national drug policy.

**Figure 1:**
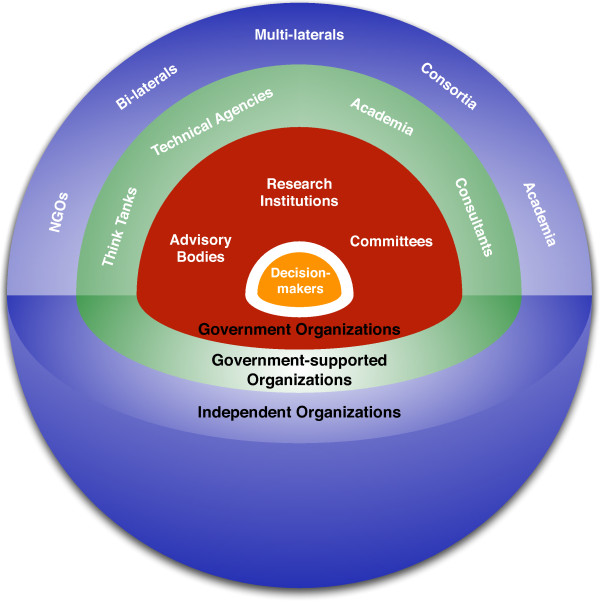
Evidence-generating organizations in LMIC health systems.

The organizational arrangement for producing research across countries can be conceptualized through a generic framework as depicted in Figure 1. Here, the different agents that produce HPSR are placed in concentric circles with decision-makers at the core. This model situates research-producing institutions in relative proximity to those making health policy decisions. The innermost ring consists of government organizations, such as special committees, research units, and advisory bodies. In this ring, actors tend to commission or actively source HPSR from the surrounding environment rather than conducting empirical HPSR themselves. They are shown outside the decision-making sphere in this diagram because their primary responsibility is to assist, not make, decisions in the health sector; however, the distinction is not always clear in some countries. The next circle consists of government-supported research organizations such as agencies, universities, think tanks, and individuals who are funded or technically assisted by the government but not directly part of it. The outer most layer consists of independent research institutions which are privately funded and managed like those belonging to multi-lateral and bi-lateral agencies, universities, NGOs, and research consortia. While proximity to decision-makers or government could increase the embeddedness of HPSR organizations, it is not necessarily the case. Decision-makers do not operate in isolation and the ring around the decision-making sphere serves as a filter for evidence absorbed into the policy process from the surrounding layers.

**Figure 2:**
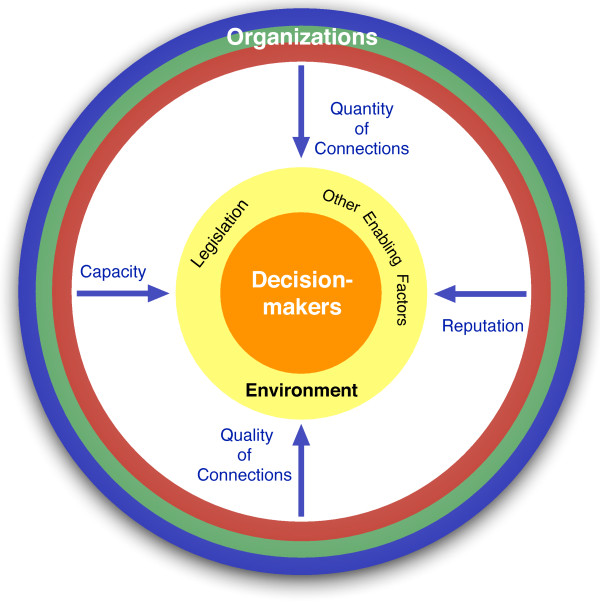
The four dimensions of embeddedness.

In Figure 2, the ring surrounding the decision-making sphere is explored in greater detail. Here, we attempt to marry the dimensions or attributes of embedded organizations in a network with the generic configuration of research organizations in LMICs (shown in Figure 1); these dimensions are essential for evidence to penetrate the decision-making sphere. The first two dimensions describe the quantity and quality of organizational connections. If a given organization has several linkages to decision-makers, as well as other organizations within the network, then it is more likely to have greater centrality and embeddedness in the network. The “quality” of these connections also matter – an organization that has links with another highly central organization in the network will possess at least as high a degree of embeddedness [13]. Also, strong links to decision-makers, or highly influential decision-makers, greatly enhance the degree to which an organization becomes embedded in the flow of evidence into policy. We discussed the third dimension earlier, when we defined “reputation” as the perception that an organization produces quality outputs for others within its domain. Reputable organizations and their products, therefore, are much more likely to be embedded and can command the attention of decision-makers. Reputable organizations may, however, produce reliable and relevant evidence in only select domains (building blocks). For this reason, we introduce the fourth dimension of capacity. Organizations that have the capacity to produce timely, accurate evidence to meet the needs of decision-makers are more likely to be embedded organizations; this type of evidence is largely HPSR. Further, we hypothesize that organizations that produce HPSR within a few given health system building block(s) tend to possess a lower degree of embeddedness than organizations that produce HPSR across more or all domains.

We present the environment surrounding decision-makers as an important mediator in the flow of HPSR to decision-making, irrespective of the organizational arrangement. For example, legislation can be an effective way of ensuring that decision-makers consider research organizations and what they produce (like in the case of Mexico). Other enabling factors specific to the policy environment include historical precedence of relying on evidence to inform policy (path-dependency), research background of decision-makers, an active civil society, a forum for consistently placing decision-makers in contact with evidence generators, well-established modes of communicating clearly between actors (policy-briefs, updates, emails, digestible reports, etc.), responsive channels for quickly sourcing evidence, and access to centrally-located HPSR generated by embedded organizations, but shared by all actors [21]. Thus, the environment is an important mediator, either hindering or facilitating the uptake of HPSR by decision-makers.

### Limitations

There are four limitations to this study. First, we make the assumption that HPSR can and should be used to influence decision-making in LMICs. We do not address equally salient normative features of the decision-making process. We recognize that decision-making is a value-laden enterprise and that the policy process is highly contextual [61], but the purpose of this paper and the model is to illustrate ways in which HPSR can better inform decision-making in LMIC health systems. Second, the analytical value of the literature review was limited by the scarcity of data on the subject. Third, the abstract nature of embeddedness posed challenges for conducting the literature review and categorizing our findings using the building blocks framework. Fourth, the study lacks a formula for translating the extracted data into a conceptual framework. The four authors collaboratively discussed the research findings and experimented with various visual representations, but were unable to develop a systematic approach. The framework presented here most accurately represents our interpretation of the literature for both the HPSR infrastructure in LMICs as well as the dimensions of embeddedness.

## Conclusions

This study raises several important considerations for both researchers and decision-makers. Embedding HPSR into decision-making processes is a context-specific process that involves multiple actors. Researchers should identify simple ways to position HPSR and HPSR organizations to address the needs of decision-makers. Researchers could test components of the conceptual framework presented in this study or use it to guide empirical and critical inquiry into the complicated processes of knowledge-translation and knowledge-utilization. The development of HPSR or “health research systems” [62], novel embedding techniques such as alignment exercises and contribution mapping [63], and linkages between health system building blocks [64] also warrant further investigation. For an organization to be embedded into the flow of information in the decision-making sphere, four key attributes must be cultivated: reputation, capacity, quality, and quantity of connections to decision-makers as well as other organizations within the health system. Decision-makers in LMICs can use this framework to a) create health policy and systems research organizations with these attributes, b) source evidence from existing organizations that possess these qualities, or c) create an environment by which these organizational qualities are allowed to flourish and HPSR can easily be absorbed into decision-making processes. Embedding HPSR into decision-making, in turn, promises to strengthen the validity of technical decisions about health and can drive overdue performance enhancements for LMIC health systems.

## Abbreviations

HPSR: Health policy and systems research; HRH: Human resources for health; LMICs: Low- and middle-income countries

## Competing interests

The authors declare that they have no competing interests.

## Authors’ contributions

ADK, KDR, NT, and AG conceived of the study and developed the study protocol. ADK conducted the literature review and drafted the manuscript. KDR revised several versions of the manuscript. NT and AG commented on successive drafts of the manuscript. All authors developed the conceptual model, read, and approved the final version of the manuscript.
